# Effects of the Aqueous Extract of *Eremomastax speciosa* (Acanthaceae) on Sexual Behavior in Normal Male Rats

**DOI:** 10.1155/2016/9706429

**Published:** 2016-07-20

**Authors:** B. Nchegang, C. Mezui, F. Longo, Z. E. Nkwengoua, A. P. Amang, P. V. Tan

**Affiliations:** ^1^Department of Animal Biology and Physiology, Faculty of Sciences, University of Yaoundé I, P.O. Box 812, Yaoundé, Cameroon; ^2^Department of Biological Sciences, Higher Teachers' Training College, University of Yaoundé I, P.O. Box 047, Yaoundé, Cameroon; ^3^Department of Organic Chemistry, Faculty of Science, University of Yaoundé I, P.O. Box 812, Yaoundé, Cameroon; ^4^Department of Biological Sciences, Faculty of Science, University of Maroua, P.O. Box 814, Maroua, Cameroon

## Abstract

*Objective*. We studied prosexual effects of* Eremomastax speciosa* aqueous extract in male adult rats.* Materials and Methods*. 100 and 500 mg/kg of extract were administered orally (days 0, 1, 4, 7, 14, and 28 (posttreatment)). The sexual behavior of rats receiving a single dose (500 mg/kg) was also evaluated after pretreatment with L*ω*-NAME (10 mg/kg), haloperidol (1 mg/kg), or atropine (5 mg/kg). Controls received distilled water or testosterone enanthate (20 mg/kg/day/3 days* (s.c.)* before the test).* Results*. The extract (days 1–14) had no significant effect on mount, intromission, and ejaculation frequencies but on day 28 (14 days after treatment), it increased frequency of mounts and intromissions at 500 mg/kg. Mount, intromission, and ejaculation latencies reduced and postejaculatory intervals decreased but the effect did not persist 2 weeks after treatment. Extract prosex effects were greatly reduced by atropine and completely abolished by haloperidol, while L*ω*-NAME increased mount latency and potentiated extract effect on intromission and ejaculation latencies.* Conclusion*. In summary,* E. speciosa* extract can have positive effects on male sexual motivation and performance when administered for two weeks at the dose of 500 mg/kg. The effects (dopaminergic and/or cholinergic dependent) tend to appear during the posttreatment period.

## 1. Introduction

The male reproductive capacity is deficient in more than 50% of infertile couples. The causes of this decline in male reproductive capacity are numerous. Sexual disorders are among the most abundant; in addition, we can mention erectile dysfunction. Erectile dysfunction is sometimes called impotence; this clinical term is retained to describe the inability to achieve and maintain penile erection sufficiently rigid to permit satisfactory sexual intercourse [1]. On the organic origin, psychogenic, erectile dysfunction is widespread and affects men of all ages (mainly 40–70 years), all occupational groups, and all sociocultural levels [2]. Diabetes, hypertension, alcoholism, smoking, and prostatic diseases are the risk factors of this sexual disorder [3]. About 150 million people worldwide suffer from erectile dysfunction [4]. This problem is also likely to seriously hamper relations within a couple sometimes leading to divorce. Because of the multifactorial etiology and investigation methods increasingly sophisticated regimens of erectile dysfunction are more and different depending on the standard of living. In developed countries, the therapy involves the implantation of penile prostheses, intracavernous injections, and the use of certain pharmaceutical products (phosphodiesterase type V inhibitors) [5]. These pharmaceuticals also favor the persistence of smooth muscle relaxation and therefore the maintenance of erection [6]. In developing countries, by cons because of the influence of the economic crisis, modern health infrastructure, high cost of drugs, and respect for customs, about 80% of the population use medicinal plants for treatment. Many plants (*Montanoa tomentosa, Syzygium aromaticum, Massularia acuminata*, and* Fadogia agrestis*) have the reputation of being an aphrodisiac. The aphrodisiac activity of these plants is attributed to the presence of one or more phytoconstituents such as sterols, phenols, alkaloids, amino acid, and saponin responsible for improving sexual function through the regulation of neurotransmitters and relaxation smooth muscle of the corpora cavernosa [7, 8]. This is the case of* E. speciosa*, widely distributed in tropical Africa [9]. The plant is a robust, polymorphous shrub growing to a height of 2 m. The stem is quadrangular, and the leaves are purple on the underside. Several constituents already have been approved, such as flavonoids, alkaloids, triterpenoids, and sterol [10].* E. speciosa* is cited for its various beneficial effects, which include stomach complaints, dysentery, hemorrhoids, urinary tract infection, painful menstruation, diarrhea, and male and female infertility [11–18] and is commonly referred to as “blood plant” since it is also widely used to treat cases of anemia. In addition to this investigation, little or no information on copulatory activity is available. The present study was designed to study the aphrodisiac activity of aqueous extract of* E. speciosa* in male rat.

## 2. Material and Methods

### 2.1. Plant Material

The fresh leaves of* Eremomastax speciosa *(Hochst.) Cufod. (Acanthaceae) were harvested between the months of August-September 2014 in Yaoundé, Cameroon political capital, specifically in common Emana and Messassi. A sample of this plant was authenticated by Paul Mezili. A specimen of this plant is kept in the National Herbarium of Cameroon, IRAD, Yaoundé, under the number HNC/136984.

### 2.2. Preparation of Water Extract

*Eremomastax speciosa* aerial parts were cut into pieces and dried outdoors in the shade at room temperature and then ground using a blender to obtain a powder. Using a spatula, 560 g of this powder was mixed in 5.6 liters of boiled water for 15 minutes. The extract solution was filtered with a filter paper (Whatman number 3). The filtrate obtained was finally dried in an oven at ventilation at 40°C. This yielded 56.20 g of dry extract, which corresponds to a yield of 10.03%.

### 2.3. Animal Material

Older adult male rats (*Rattus norvegicus*) of 3.5–4 months, weighing between 200 and 230 g, were used for the experiments on copulatory activity. These animals (males and females) were from the animal facility of the Faculty of Sciences of the University of Yaounde I, Cameroon. They were raised in rooms at room temperature. In these premises, the photoperiod was 12 hours and humidity 50%. The animals had ad libitum access to water and food (66.87% corn flour, 30.85% fish meal, 1.03% shell powder, 1.03% of salt, 0.11% peanut oil, and 0.11% of multivitamin). The ethics committee of the Cameroon Ministry of Scientific Research and Innovation (MINRESI) approved all experimental procedures.

### 2.4. Animal Grouping and Extract Administration

24 rats gonado-intact and sexually experienced were randomly assigned to one of the following groups: group 1 receiving distilled water (1 mL/kg) and serving as a neutral control group, group 2 or standard group treated by a subcutaneous injection of testosterone enanthate (20 mg/kg/day) for 3 days before the start of copulatory tests [19], and groups 3 and 4, respectively, treated by the aqueous extract of* E. speciosa* at doses of 100 and 500 mg/kg. In rats, distilled water and the extract were orally administered once a day between 20 h and 20 h 30 local time and for a period of 14 days. On days 0, 1, 4, 7, and 14 of treatment and day 28 (posttreatment), the sexual behavior of animals was analyzed in a quiet enclosure for one hour.

In a complementary study, the impact of a dose of 500 mg/kg extract of* E. speciosa* on dopaminergic, cholinergic, and nitergic systems was studied. 30 gonado-intact rats were used in this study and divided into 6 groups of 5 animals each. Prior to administration of the extract of* E. speciosa* (500 mg/kg), the rats received, as appropriate, intramuscular injection of atropine (5 mg/kg) (CC Pharma, Belgium) or haloperidol (1 mg/kg) (Janssen-Cilag, France) or an intraperitoneal injection of L*ω*-NAME (10 mg/kg) (Sigma, USA) 1 h before the coupling test. The doses of these substances were chosen based on preliminary work. Control animals were treated with diluents solvent (5 mL/kg of Tween 80; 0.25% in 0.9% NaCl) intraperitoneally or intramuscularly.

### 2.5. Male Rat Sexual Behavior: Test Procedure

After 30 minutes of acclimatization of each male in copulation cage, an ovariectomized female [20] made receptive by subcutaneous and sequential injections of 30 micrograms of estradiol benzoate (Sigma, USA) and 500-microgram progesterone (Bayer Pharma AG, Germany) 48 pm and 6 am, respectively, before the start of the test, was introduced in the cage. The following copulatory parameters were recorded for 1 h with reference to standard methods [21–23]: mounted frequency (FM) or the total number of mounts during the observation period; frequency interferences (FI) or the total number of interferences during the hour of study; frequency of ejaculations (FE) which is the total number of ejaculations; mount latency (ML) and intromission latency (IL) which are the time from intromission of a female in the cage and the first intromission and mounts, respectively; ejaculation latency which is the time between the first ejaculation and the first intromission; postejaculatory interval which is the time between the first ejaculation and the new intromission. Some additional male sexual behavior parameters computed include % mounted = (number mounted/number paired) × 100; % intromitted = (number of rats that intromitted/number paired) × 100; % index of libido = (number mated/number paired) × 100; % ejaculation = (number of rats that ejaculated/number paired) × 100; copulatory efficiency = (IF divided by MF + IF) × 100; inter-copulatory efficiency = average time between intromissions (calculated as ejaculated latency divided by intromission frequency).

### 2.6. Statistical Analysis

The statistical analysis was performed using the GraphPad Prism software. The Student test Newman Keul was selected for the comparison of averages. The difference between two is said to be significant if *P* < 0.05.

## 3. Results

### 3.1. Prosexual Effects of* E. speciosa* Aqueous Extract on Some Parameters of Performance and Sexual Motivation in Gonad-Intact Male Rats

Treatment with the aqueous extract of* E. speciosa* (days 1–14) had no statistically significant effect on the frequencies of mounts, intromissions, and ejaculations compared with animals treated with distilled water. However, on day 28 (14 days posttreatment), there were significant increases in the frequency of mounts and of intromissions at the 500 mg/kg dose of extract compared with significant distilled water controls (*P* < 0.05) and the positive controls (*P* < 0.01) (Tables 1 and 2). Ejaculation frequencies remained unchanged up to day 14 of extract treatment and increased to 3.66 ± 0.61 and 4.20 ± 0.66 on day 28 compared with day 0 observations (2.00 ± 0.51 and 3.83 ± 0.54) (Table 3). On day 7, there were significant decreases in the mount latency, intromission latency, and ejaculation latency in rats receiving the extract at doses of 100 and 500 mg/kg (*P* < 0.05–*P* < 0.001) compared with day 0 and neutral control values (Tables 4, 5, and 6). On day 28 (posttreatment) mount latency decreased in rats given the extract and testosterone compared with day 0. Postejaculatory intervals decreased only for the 100 mg/kg dose on day 14 of treatment compared with days 0–7 but the effect did not persist 2 weeks after cessation of treatment (Table 7). The computed male sexual behavior parameters including percentages of intromissions and ejaculations and index of libido were higher in the extract-treated animals compared with the distilled water controls. In contrast, the extract reduced intercopulatory efficiency of the treated animals compared with the control animals. Copulatory efficiency decreased in rats receiving the extract at the dose of 100 mg/kg compared with the neutral controls (Table 8).

### 3.2. Effects of Haloperidol, Atropine, or L*ω*-NAME Pretreatment upon the Prosexual Effect Induced by the Aqueous Extract of* E. speciosa *(500 mg/kg)

**Figure 1:**
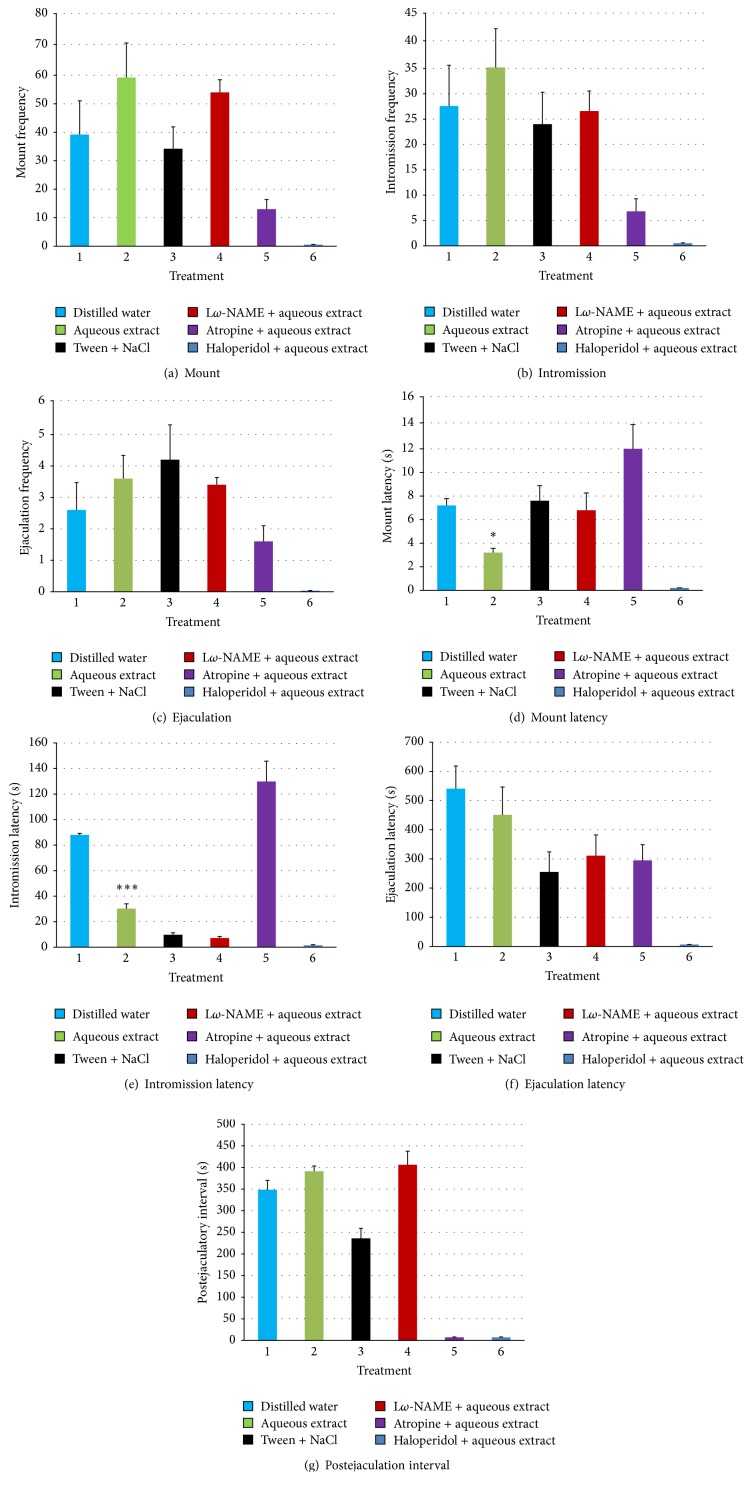
Effects of the aqueous extract (500 mg/kg) of* E. speciosa* on the sexual behavior of sexually experienced rats pretreated with L*ω*-NAME (10 mg/kg), haloperidol (1 mg/kg), or atropine (5 mg/kg). ^*∗*^ *P* < 0.05; ^*∗∗∗*^ *P* < 0.001 versus control neutral (distilled water).

The intraperitoneal injection of L*ω*-NAME (10 mg/kg) before administration of aqueous extract of* E. speciosa* (500 mg/kg) had no influence on the frequency of mounts, intromissions, and ejaculations. Pretreatment with L*ω*-NAME increased mount latency by 50%, potentiated the effect of extract (decrease) on intromission and ejaculation latencies, but did not influence extract effect on postejaculatory interval. However, pretreatment with atropine (5 mg/kg) significantly reduced all the copulatory parameters while haloperidol (1 mg/kg) pretreatment completely abolished them (Figures 1(a)–1(g)).

## 4. Discussion

The effects of* E. speciosa* extract on libido were studied by measuring some sexual performance parameters in intact adult male rats. The results show that the aqueous extract of* E. speciosa* stimulated sexual function of male rats. Although extract treatment for 14 consecutive days tended to increase the number of mounts, intromissions, and ejaculations compared with the neutral controls, the differences were not statistically significant. However, the frequencies of mount and intromission increased significantly 2 weeks (especially at dose 500 mg/kg) following stoppage of treatment. On the other hand, mount, intromission, and ejaculatory latency reduced significantly by day 7 of extract treatment, but these effects did not persist 2 weeks following cessation of treatment. Although classical measurements of erection frequency were not performed in this study, both mount and intromission frequencies, which are useful indices of vigor, libido, and potency [24], increased during and after end of extract treatment. Thus, while increased mount frequency is an indication of heightened sexual motivation, increased intromission frequency is an indicator of the efficiency of erection, penile orientation, and increased activation of ejaculatory reflexes by the extract [25]. Increased mount and intromission latencies are thus suggestive of improved libido possibly due to increased anterior pituitary hormones that stimulate dopamine receptor synthesis [26–28]. This increase in sexual performance parameters in rats treated with* E. speciosa* extract would be due to the action of the active ingredients therein. Phytochemical studies of the aqueous extract [10] have revealed the presence of phytosterols and flavonoids. Phytosterols are substances with known prosexual effects [29]. Flavonoids are involved in the onset of penile erection and improved sexual performance [30, 31]. Subcutaneous injection of testosterone enanthate (20 mg/kg/day) in normal rats for 3 days before the start of copulatory tests has been shown to significantly reduce the latent period of erection (the time to reach full erection following the start of electrical stimulation) [19] and to increase frequency of erections [32]. In our study, in rats treated with testosterone, sexual performance parameters (frequency of ejaculations and of intromission) were high compared to extract-treated groups and neutral control. Indeed, it has been shown that the administration of testosterone increases copulatory ability in rats [25]. Copulatory behavior in rats is activated by dihydrotestosterone (DHT) and estradiol from the aromatization of testosterone in the brain [33–35]. After the posttreatment study, copulatory parameters indicative of increased libido (mount and intromission frequencies) in rats treated with 500 mg/kg dose increased compared with day 14 observations. These observations suggest possible effects of slowly accumulated secondary metabolites synthesized over time which may affect hormones and receptor synthesis or receptor binding ability. Diabetes mellitus is a risk factor for erectile dysfunction [36], and long-term use of histamine H_2_ receptor antagonists (e.g., Cimetidine) in gastroduodenal ulcer treatment leads to reduced libido.* E. speciosa* has been cited for its ethnopharmacological application in diabetes management [14] and other workers [10, 37] have reported the cytoprotective and antisecretory effects of* E. speciosa* extract in peptic ulcer management. Therefore* E. speciosa* extract may have possible long-term benefits in sustaining increased levels of libido especially when used to treat conditions that represent risk factors for erectile dysfunction.

Mount latency (ML) and intromission latency (IL) are indicators of sexual motivation. ML and IL are inversely proportional to sexual motivation. Therefore, the decrease in the mount and intromission latencies observed in this study on days 1, 7, and 14 at the doses of 100 and 500 mg/kg of extract might imply stimulation of sexual motivation and arousability. It may also be an indication of enhanced sexual appetitive behavior in male rats. Furthermore, the reduced ejaculation latency at all the doses on day 7 could be an indication of enhanced copulatory performance. In addition, the display of pelvic thrusting during intromission and ejaculation by the extract-treated animals in this study further indicated that the male copulatory organ was in contact with the vaginal orifice which might have activated or strengthened lordosis in the female rats. A postejaculatory interval (PEI) of more than 5400 seconds indicates that the male is sexually exhausted and the intensity of sexual behavior will be reduced in subsequent mating [37]. In addition, reduction of the postejaculatory interval (PEI) is also an indication of sustained increase in sexual activity [38].

Effects of the extract were also evaluated in the presence of L*ω*-NAME (an inhibitor of nitric oxide synthase), haloperidol (a nonspecific inhibitor of dopamine receptors), and atropine (a muscarinic receptor inhibitor) in order to detect the possible route of action of the bioactive principles. Pretreatment with L*ω*-NAME triggered sexual desire by reducing intromission and ejaculation latencies. This would suggest the noninvolvement of nitric oxide in the development of prosexual effects of the extract. Nitric oxide is a mediator in the smooth muscle relaxing action. It is generated from L-arginine by nitric oxide synthase enzyme present in endothelial cells (eNOS) and corpora cavernosa of the penis in neurons. NO, an established mediator of penile erection, plays an important role in sensory nerves that stimulate copulatory activity. It stimulates the relaxation of penile vasculature and trabecular smooth muscle essential for penile erection [39, 40]. Pretreatment with atropine (a muscarinic receptor antagonist) or haloperidol (a dopamine receptor antagonist) resulted in the suppression of prosexual effects of the extract. This suggests the involvement of cholinergic receptors and dopamine receptors in the induction of prosexual effects by the extract. Dopamine, synthesized in the medial preoptic area (MPOA) and in many areas of the central and peripheral nervous system, facilitates sexual activity in male rats. The stimulation of dopamine receptors in the MPOA facilitates copulation, sexual motivation, and genital reflex in male rats. The relaxing action of acetylcholine in erectile function may be related to its interaction with muscarinic receptors in the central area and the hypothalamus [41, 42].

A study by others [43] has shown that the dose of 500 mg/kg of* E. speciosa* aqueous extract is nontoxic in acute and subacute toxicity study in rats. The observed effects of the extract on male sexual behavior may be attributed to the presence of bioactive compounds (phytosterols and flavonoids) [10] with well-known sexual stimulant properties [44]. Other workers [28, 45] attributed the increased sexual performance (following treatment with* Massularia acuminata* extract and* Tricholepis glaberrima* extract, resp.) to the androgenic and gonadotropic properties of flavonoids and their antioxidant effects. Flavonoids and sterols can stimulate endogenous testosterone levels probably by raising the levels of luteinizing hormones [46, 47]. Aphrodisiacs are required to improve male sexual function under stressful conditions, and the extract of* Moringa oleifera* has been shown to enhance sexual performance in stressed rats possibly through increased numbers of interstitial cells of Leydig and spermatozoa [48]. The aphrodisiac effects of* E. speciosa* extract may be enhanced by its antioxidant activity which has earlier been reported by previous workers [36].

## 5. Conclusion

The results of the present study show that the extract of* E. speciosa* can have positive effects on male sexual motivation and performance when administered for two weeks at the dose of 500 mg/kg. The effects of the extract, which may be attributed to its phytochemical constituents, tend to appear during the posttreatment period and would justify the use of* E. speciosa* as a sexual stimulant.

## Figures and Tables

**Table 1:** Effects of *E. speciosa* extract on the frequency of mounts of normal male rats.

Treatment	Day of observation					
	D0	D1	D4	D7	D14	D28
H_2_O (1 mg/kg)	43.33 ± 7.03	48.66 ± 14.93	42.00 ± 12.39	58.00 ± 12.44	42.50 ± 6.57	44.50 ± 10.04
Testosterone (20 mg/kg)	57.16 ± 9.76	73.16 ± 9.65	56.50 ± 4.55	58.50 ± 7.98	70.00 ± 12.56	46.33 ± 6.92
Extract (100 mg/kg)	88.83 ± 3.44^*∗∗β*^	70.00 ± 7.23	49.00 ± 16.39	66.83 ± 13.38	77.16 ± 22.63	72.16 ± 9.27
Extract (500 mg/kg)	78.83 ± 11.27^*∗*^	81.00 ± 18.06	59.66 ± 12.88	61.16 ± 6.90	56.00 ± 8.45	84.60 ± 6.02^*∗β*^

Values are expressed as mean ± SEM (*N* = 6). In the same column, ^*∗*^ *P* < 0.05; ^*∗∗*^ *P* < 0.01 versus negative control and ^*β*^ *P* < 0.05 versus testosterone control (frequency = number).

**Table 2:** Effects of *Eremomastax speciosa* extract on the intromission frequency of the gonado-intact male rats.

Treatment	Day of observation					
	D0	D1	D4	D7	D14	D28
H_2_O (1 mg/kg bw)	23.00 ± 5.92	23.66 ± 8.06	24.66 ± 7.54	35.16 ± 7.53	29.00 ± 6.43	25.66 ± 5.93
Testosterone (20 mg/kg)	21.50 ± 6.71	47.00 ± 8.14	35.66 ± 3.48	37.50 ± 4.93	47.16 ± 7.48	31.33 ± 4.58
Extract (100 mg/kg)	26.16 ± 1.35	35.66 ± 7.60	33.66 ± 12.14	39.00 ± 7.99	43.83 ± 11.36	43.50 ± 5.88
Extract (500 mg/kg)	21.66 ± 3.50	36.66 ± 9.91	27.00 ± 6.76	32.33 ± 5.44	36.83 ± 5.26	52.40 ± 3.93^*∗β*^

Values are expressed as mean ± SEM (*N* = 6). In the same column, ^*∗*^ *P* < 0.05 versus negative control and ^*β*^ *P* < 0.05 versus testosterone control (frequency = number).

**Table 3:** Effects of the *E. speciosa* extract on the frequency of ejaculations of gonado-intact rats.

Treatment	Day of observation					
	D0	D1	D4	D7	D14	D28
H_2_O (1 mg/kg)	2.50 ± 0.76	1.83 ± 0.70	1.50 ± 0.56	1.66 ± 0.55	2.33 ± 0.61	2.50 ± 0.56
Testosterone (20 mg/kg)	2.16 ± 0.74	4.00 ± 0.36	2.66 ± 0.42	3.00 ± 0.36	3.16 ± 0.79	3.50 ± 0.84
Extract (100 mg/kg)	2.00 ± 0.51	3.33 ± 0.49	1.83 ± 0.70	2.50 ± 0.67	2.33 ± 0.71	3.66 ± 0.61
Extract (500 mg/kg)	3.83 ± 0.54	2.83 ± 0.65	1.83 ± 0.47	1.66 ± 0.21	3.33 ± 0.49	4.20 ± 0.66

Values are expressed as mean ± SEM (*N* = 6). Frequency = number.

**Table 4:** Effects of the extract of *E. speciosa* on the latency of mounts in gonado-intact male rats.

Treatment	Day of observation					
	D0	D1	D4	D7	D14	D28
H_2_O (1 mg/kg)	20.16 ± 2.19^b^	15.00 ± 1.61^a^	10.16 ± 0.16^b^	14.33 ± 1.82^b^	6.83 ± 0.79^b^	8.83 ± 0.94^b^
Testosterone (20 mg/kg bw)	17.00 ± 0.77^a^	9.83 ± 1.64^*∗∗∗*b^	9.00 ± 0.85^b^	14.00 ± 0.68^ab^	10.16 ± 1.49^*∗*a^	12.16 ± 1.57^b^
Extract (100 mg/kg)	17.00 ± 1.67^a^	8.00 ± 1.34^*∗∗∗*b^	11.50 ± 0.67^*β*cb^	1.33 ± 0.33^*∗∗∗βββ*d^	1.66 ± 0.21^*∗∗∗βββ*ed^	9.66 ± 2.26^fd^
Extract (500 mg/kg)	12.50 ± 1.33^a^	14.33 ± 1.34^*∗∗∗*ba^	6.50 ± 0.42^*∗∗∗ββ*c^	2.00 ± 0.36^*∗∗∗βββ*d^	9.16 ± 0.30^eca^	8.16 ± 2.12^fca^

Values are expressed as mean ± SEM (*N* = 6). ML (seconds); different superscript letters in the same row refer to a significant difference; in the same column, ^*∗*^ *P* < 0.05; ^*∗∗∗*^ *P* < 0.001 versus neutral control and ^*β*^ *P* < 0.05; ^*ββ*^ *P* < 0.01; ^*βββ*^ *P* < 0.001 versus testosterone control.

**Table 5:** Effects of *Eremomastax speciosa* extract on the intromission latency of gonado-intact male rats.

Treatment	Day of observation					
	D0	D1	D4	D7	D14	D28
H_2_O (1 mg/kg)	20.33 ± 3.25^a^	18.00 ± 0.77^ba^	13.83 ± 0.65^cb^	21.83 ± 1.55^da^	10.83 ± 1.62^ec^	11.33 ± 1.08^fc^
Testosterone (20 mg/kg bw)	35.16 ± 5.81^a^	10.33 ± 2.33^b^	16.16 ± 1.07^b^	13.00 ± 1.94^*∗∗∗*b^	11.50 ± 1.05^b^	13.16 ± 1.27^b^
Extract (100 mg/kg)	24.83 ± 1.51^a^	15.33 ± 3.92^b^	15.83 ± 1.72^b^	11.00 ± 1.43^*∗∗∗*b^	11.33 ± 1.30^b^	13.66 ± 2.26^b^
Extract (500 mg/kg)	33.83 ± 9.62^a^	16.50 ± 2.59^b^	18.16 ± 1.60^b^	10.66 ± 1.40^*∗∗∗*b^	7.00 ± 0.81^b^	8.20 ± 2.59^b^

Values are expressed as mean ± SEM (*N* = 6). IL (seconds); different superscript letters in the same row refer to a significant difference; in the same column, ^*∗∗∗*^ *P* < 0.001 versus neutral control.

**Table 6:** Effects of *E. speciosa* extract on the ejaculation latency of gonado-intact rats.

Treatment	Day of observation					
	D0	D1	D4	D7	D14	D28
H_2_O (1 mg/kg)	301.33 ± 79.74^ab^	291.33 ± 6.87^b^	588.33 ± 43.01^c^	1056.33 ± 57.01^d^	309.66 ± 40.94^eb^	400.00 ± 53.12^fb^
Testosterone (20 mg/kg)	356.83 ± 39.22^ab^	367.00 ± 12.84^*∗*ab^	425.50 ± 86.20^ab^	397.33 ± 60.25^*∗∗∗*ab^	574.83 ± 56.13^*∗∗*a^	303.33 ± 51.07^b^
Extract (100 mg/kg)	591.50 ± 58.57^*∗∗β*a^	378.16 ± 29.67^*∗*ab^	552.00 ± 81.21^ab^	325.00 ± 86.20^*∗∗∗*b^	619.83 ± 22.86^*∗∗*ac^	491.16 ± 57.87^ab^
Extract (500 mg/kg)	267.83 ± 42.24^a^	622.16 ± 26.59^*∗∗∗βββ*b^	678.16 ± 50.99^cb^	680.00 ± 31.64^*∗∗∗βββ*dcb^	254.16 ± 47.28^*βββ*ea^	309.16 ± 23.90^fa^

Values are expressed as mean ± SEM (*N* = 6). EL (seconds); different superscript letters in the same row refer to a significant difference; in the same column, ^*∗*^ *P* < 0.05; ^*∗∗*^ *P* < 0.01; ^*∗∗∗*^ *P* < 0.001 versus neutral control and ^*β*^ *P* < 0.05; ^*βββ*^ *P* < 0.001 versus testosterone control.

**Table 7:** Effects of *E. speciosa* extract on the postejaculatory interval of gonado-intact rats.

Treatment	Day of observation					
	D0	D1	D4	D7	D14	D28
H_2_O (1 mg/kg bw)	331.20 ± 28.06^aef^	277.00 ± 26.48^ba^	434.00 ± 16.56^c^	532.33 ± 29.32^d^	386.16 ± 15.88^ec^	379.16 ± 19.94^fc^
Testosterone (20 mg/kg)	351.00 ± 32.96^ab^	406.16 ± 36.12^*∗∗*abc^	505.50 ± 23.65^c^	459.33 ± 36.66^abc^	345.50 ± 36.82^b^	345.16 ± 27.48^a^
Extract (100 mg/kg)	390.16 ± 34.42^ab^	461.00 ± 26.92^*∗∗∗*b^	484.33 ± 29.13^cb^	264.00 ± 36.05^*∗∗∗ββ*def^	202.16 ± 26.28^ea^	294.33 ± 26.70^fa^
Extract (500 mg/kg)	365.00 ± 30.73^a^	464.33 ± 19.71^*∗∗∗*b^	571.00 ± 2.58^*∗∗∗β*c^	649.33 ± 44.78^*∗ββ*dc^	435.66 ± 31.74^abe^	352.00 ± 18.88^af^

Values are expressed as mean ± SEM (*N* = 6). IPE (seconds); different letters in the same row refer to a significant difference; in the same column, ^*∗*^ *P* < 0.05; ^*∗∗*^ *P* < 0.01; ^*∗∗∗*^ *P* < 0.001 versus neutral control and ^*β*^ *P* < 0.05; ^*ββ*^ *P* < 0.01 versus testosterone control.

**Table 8:** Effect of aqueous extract of *E. speciosa* for 14 days on computed male rat sexual behavior parameters.

Parameter	Neutral control	Positive control	Extract (mg/kg bw)	
	(1 mL/g)	(20 mg/kg)	100	500
% mounted	100	100	100	100
% intromitted	100	100	100	100
% index of libido	83.33	100	100	100
% ejaculation	83.33	100	83.33	100
Copulatory efficiency (%)	42.16 ± 0.49	40.62 ± 1.66	38.05 ± 1.45	40.05 ± 1.56
Intercopulatory efficiency (sec)	26.22 ± 2.53	22.04 ± 1.96	21.61 ± 91.44	16.06 ± 1.46^*∗∗*^

Values for copulatory efficiency and intercopulatory efficiency are expressed as mean ± SEM (*N* = 6).

^*∗∗*^ *P* < 0.01; statistically significant compared with neutral control.
